# The ASCO Study of Collaborative Practice Arrangements: A Physician Assistant’s Perspective

**DOI:** 10.6004/jadpro.2012.3.1.5

**Published:** 2012-01-01

**Authors:** Alicia C Ross

**Affiliations:** From The University of Texas M.D. Anderson Cancer Center, Houston, Texas

One in two men and women will be diagnosed with cancer of all types during their lifetimes, based on lifetime risk rates from 2005 through 2007; 1 in 6 men with prostate cancer, 1 in 8 women with breast cancer, 1 in 14 men and women with lung and bronchus cancer, and 1 in 20 men and women with colorectal cancer. It was estimated that 1,529,560 men and women would be diagnosed with, and 569,490 men and women would die from cancer in 2010 (National Cancer Institute, 2011). Cancer is the second leading cause of death, close behind only heart disease (Centers for Disease Control and Prevention, 2007). As health-care providers, it is certain that we will all encounter patients diagnosed with cancer, patients receiving active therapy for cancer, and/or patients undergoing clinical surveillance for a history of cancer, regardless of our specialty.

The physician assistant (PA) profession is estimated to grow 39% from 2008 to 2018, faster than the average for all occupations (United States Bureau of Labor, 2011). The American Association of Physician Assistants (AAPA) 2010 census reported an increase to 15.2% of PAs working in oncology. This is strikingly different than the less than 2.5% reported in 2008 and 2009 AAPA census data. Could this increase indicate that more PAs are going into oncology? Potentially, but this was the first year that the AAPA census performed a validation study, which results in more accurate data but unfortunately cannot be compared to prior results head to head (AAPA, 2010). What do we really know about PAs and their role in oncology settings?

## Workforce Shortages

In March 2007, Erikson, Salsberg, Forte, Bruinooge, & Goldstein reported the results of a study undertaken by the American Society of Clinical Oncology (ASCO) Board of Directors estimating a 48% rise in demand for oncologic services between 2005 and 2020. Interestingly, the supply of services provided by oncologists during this time was only expected to rise by 14%, not meeting the anticipated demand. A variety of scenarios were presented to attempt to meet this demand, including increased use of nurse practitioners (NPs) or PAs. According to the practitioner survey, more than half (54%) of the oncologists were already working with NPs/PAs, and averaged higher weekly rates than those who did not. In addition, productivity was highest when utilizing NPs/PAs for advanced activities, such as assisting with new patient consults, ordering routine chemotherapy, and performing invasive procedures. Results also suggested those physicians working with NPs/PAs felt they had improved efficiency and patient care as well as professional satisfaction.

Despite these positive outcomes, it was concluded that the number of NPs/PAs may not be able to meet this rising demand (Erikson et al., 2007). Prior to this 2007 ASCO Workforce Study, only a few studies or editorials had been written with regard to nonphysician practitioners (NPPs) in oncology, and it seemed there was little interest. However, with this suggestion that NPPs could potentially help meet the demand of oncology services anticipated by 2020, a rising interest in understanding the role of NPPs in oncology emerged. Questions began to arise regarding whether the impact of adding NPPs to an oncology practice may have been substantially underestimated by the workforce study as more focus was directed to weekly encounter rates vs. looking at the productivity seen when providing direct patient care (Polansky, Ross, & Coniglio, 2010).

## Understanding the Workplace

To further understand the practice models between oncologists and NPPs, the ASCO Study of Collaborative Practice Arrangements (SCPA) was created. In September 2011, Towle et al. reported results from a national survey looking at 226 practices in 43 states, and then a more detailed look at a study group that included 33 practice sites in 24 states; 27 sites completed the study. The practice selection strived to include a variety of practice sizes, structures, and geographic locations, as well as the employment of NPPs. The study concluded that once again there was high satisfaction among physicians and NPPs, but also reported high patient satisfaction with the collaborative practice model. It was shown that most (98%) patients acknowledged they were seeing a NPP, and NPPs working with all practice physicians resulted in higher productivity (Towle et al., 2011).

The ITPM (incident-to practice model) was the prevalent model in the survey and study groups, in which the NPP routinely sees patients independent of the physician, but follows a care plan developed by the physician and consult physician if necessary. However, in both the survey group and the study group, the majority of respondents were from physician-owned private practices. This is contrary to another recent study by Hinkel et al. (2010) that included 15 National Cancer Center Network (NCCN) member institutions when striving to better define how cancer centers utilize NPPs and to establish better productivity benchmarks. This basic difference in the surveyed populations may bring light to the fact that NPPs’ roles in the collaborative model in private practice may be different than those of NPPs employed at cancer centers. Does this matter? When it comes to providing good health care, the answer is probably "no." However, when trying to understand the collaborative practice models across the nation to reach conclusions that could establish a nationwide collaborative practice model going forward, it is important to look at the environment in which these results are gathered and assess whether it is applicable to the entire population.

As more studies are being undertaken, positive feedback is unfolding regarding the utilization of NPPs in the oncologic setting. We have a better understanding of the roles of NPPs, successful collaborative methods, and ways to maximize reimbursement, but some basic questions still remain. How do we better educate the NPPs to be prepared for a role in oncology, and how do we continue to convince still-debating physicians that hiring an NPP will be a worthwhile decision?

## Training Needs

Advanced oncology programs are available for both NPs and PAs, but most NPPs require "on-the-job training" or practice-based training. A study undertaken evaluating the roles of PAs in 2009 reported that out of 154 PAs in an academic setting, 91.4% indicated that physician mentorship was of "great importance for obtaining their knowledge base" (Ross, Polansky, Parker, & Palmer, 2010). Furthermore, when asked how long it took to become "fully competent in the practice of oncology within a setting and discipline," 86% reported 6 months to 2 years, with the majority (61%) of those PAs reporting 1 to 2 years. Therefore, despite fairly quick acquisition of knowledge with no prior specialty training, physicians must understand their role in the education of the NPP. Goldstein stated that "oncologists will have to be team leaders" when incorporating NPPs into the practice. However, he also stated that the lack of oncology experience and training are challenges (Goldstein et al., 2008).

Current efforts are being undertaken to establish better educational modules and training programs for PAs in the workplace to perhaps decrease the time it takes to become fully competent in oncology (Polansky, 2011). As we try to educate physicians about the benefits of incorporating NPPs into their practice, there will continue to be those who question the benefit or more likely, debate the hassle. The SCPA demonstrated that some physicians indicated they did not employ NPPs because "physicians are not interested in working with NPPs," "we do not have the patient volume to support an NPP," and "we have worked with NPPs in the past and it didn’t work out" (Towle et al., 2011). It would be interesting to question these physicians further to learn what type of collaborative practice arrangement was in place, the number of years experience of the NPP, the time required to train the NPP, and the knowledge of the physician with regard to the scope of practice of an NPP and their expectations. Continued work needs to be done in better preparing NPPs for a job in oncology as well as ensuring that NPPs are being utilized in an advanced capacity; otherwise the productivity and satisfaction yield may be low.

## Recruiting PAs to Oncology

What else can we do to help meet the demand? Recruit, recruit, recruit. Recruiting PAs to the field of oncology continues to be a necessity. How do we do it? First and foremost, PAs need to be exposed to oncology in their training. Polansky & Kowis (2010) conducted a small study surveying PA students that had completed > 8 months of clinical training (64%) prior to entering an oncology rotation. Most students reported that they received > 6 hours of instruction in cancer prevention and the majority reported having < 6 hours of instruction in cancer pathology, cancer treatment, and supportive/end-of-life care (Polansky & Kowis, 2010).

What else can we do to help meet the demand? Recruit, recruit, recruit. Recruiting PAs to the field of oncology continues to be a necessity. How do we do it? First and foremost, PAs need to be exposed to oncology in their training. Polansky & Kowis (2010) conducted a small study surveying PA students that had completed > 8 months of clinical training (64%) prior to entering an oncology rotation. Most students reported that they received > 6 hours of instruction in cancer prevention and the majority reported having < 6 hours of instruction in cancer pathology, cancer treatment, and supportive/end-of-life care (Polansky & Kowis, 2010).

There needs to be more focus on exposing PA students to basic diagnosis, workup, staging, referral process, and long-term care and surveillance of cancer patients while in school. This improvement in training has two potential significant benefits within oncology and primary care. First, new hire PAs will have a higher level of competency in the field of oncology at the onset, and therefore will reduce the burden on the attending physician(s) and shorten the amount of practice-based training required, leading to a more efficient, productive, and high-satisfaction practice. Second, the ASCO Workforce Study also proposed that increasing the role of primary care providers could help in alleviating the shortage of oncologists. Physician assistants that choose to work in primary care and internal medicine subspecialties (41% of PAs in the AAPA census) will have better training to prepare them to see this cancer patient population. With these potential benefits, further interest in looking into PA programs and incorporating better oncology training is underway.

## Future Directions

Physician assistants can help meet the demands of the predicted shortage of oncologic services. We are beginning to better understand the role of PAs, but there is room for improvement. We need to better understand the collaborative practice model and how that may differ from large academic cancer institutions to private practice. We need to establish an educational pathway for PAs when entering an oncologic practice to help with immediate integration into the practice to minimize any anticipated burden on the physician. We need to expose PA students to oncology during their graduate training and meet with PA programs to encourage a higher percentage of encounters with cancer patients during their training. This last goal is the most promising, as it would not only assist in a more prepared PA entering the field of oncology, but would prepare all PAs entering the medical field, as we will rely on our partners in primary care, internal medicine, palliative medicine, and hospice to help meet the rising demand.

Cancer is a problem; it is the second leading cause of death and we will all be faced with evaluating, treating and/or following patients with cancer, regardless of the specialty we choose. PAs can make a difference, but some changes must be made to properly prepare PAs for the task that lies ahead.
